# Reproducibility of measurements on physical performance in head and neck cancer survivors; measurements on maximum mouth opening, shoulder and neck function, upper and lower body strength, level of physical mobility, and walking ability

**DOI:** 10.1371/journal.pone.0233271

**Published:** 2020-09-03

**Authors:** Gerben van Hinte, Ruud A. Leijendekkers, Bram te Molder, Lizzy Jansen, Corinda Bol, Matthias A. W. Merkx, Robert Takes, Maria W. G. Nijhuis-van der Sanden, Caroline M. Speksnijder

**Affiliations:** 1 Department of Rehabilitation, Radboud University Medical Center, Nijmegen, The Netherlands; 2 Orthopaedic Research Laboratory, Radboud Institute for Health Sciences, Radboud University Medical Center, Nijmegen, The Netherlands; 3 Radboud Institute for Health Sciences, IQ Healthcare, Radboud University Medical Center, Nijmegen, The Netherlands; 4 Research Group Musculoskeletal Rehabilitation, HAN University of Applied Sciences, Nijmegen, The Netherlands; 5 Department of Oral and Maxillofacial Surgery, Radboud University Medical Center, Nijmegen, The Netherlands; 6 Department of Otorhinolaryngology, Head and Neck Surgery, Radboud University Medical Center, Nijmegen, The Netherlands; 7 Department of Head and Neck Surgical Oncology, University Medical Center Utrecht Cancer Center, Utrecht University, Utrecht, The Netherlands; 8 Department of Oral and Maxillofacial Surgery and Special Dental Care, University Medical Center Utrecht, University of Utrecht, Utrecht, The Netherlands; University of Florida, UNITED STATES

## Abstract

**Background:**

Survivors of Head and Neck Cancer experience specific problems in functional performance. The aim of this study was to obtain the test-retest reliability of measurements on Maximal Mouth Opening (MMO), shoulder and neck function, lower and upper body strength, level of mobility and walking ability.

**Materials and methods:**

Test-retest study design. Measurements on MMO (intra- and extra orally), Active range of motion of shoulders and neck, 30 Seconds Chair Stand Test, Grip Strength, Timed Up and Go test, and Six Minute Walk test.

**Results:**

In total 50 participants were included. The mean age was 68.6. ± 9.9 years and median time since end of treatment was 3.0 years (Q1–Q3: 1.0–5.25 years). We found good to excellent test-retest reliability on the core set of measurements (Intraclass Correlation Coefficient (ICC) 0.77 to 0.98). Measurement of MMO with cardboard card, forward flexion shoulder and Six Minute Walk test had a relatively small measurement error (Smallest Detectable Change (SDC) % 5.4% - 15.1%). Measurement of MMO with a caliper, shoulder abduction, shoulder external rotation, later flexion and rotation of the neck, grip strength, 30 Seconds Chair Stand Test, and Timed up and Go test had a relatively large measurement error (SDC% 19.8% - 44.7%).

**Conclusion:**

This core set of measurements on physical performance is found reliable and therefore able to differentiate in physical performance. The reported measurement errors should be taken into consideration when interpreting the results of repeated measurements.

**Implications for cancer survivors:**

A core set of physical measurements can be used to measure physical performance in survivors of Head and Neck Cancer.

## Introduction

Curative treatment of Head and Neck Cancer (HNC) may consist of surgery, radiotherapy, chemotherapy, or a combination of these treatments. The choice and extent of treatment is influenced by tumor size and cervical lymph node involvement expressed in TNM-status [1]. Survivors of HNC (sHNC) commonly experience treatment-related morbidity that impairs their physical, social, emotional, and psychological performance [2, 3].

Local morbidity can be related to alterations in the functional anatomy and physiology of the head and neck. Local limitations can occur in the Maximum Mouth Opening (MMO) and other oral functions (speech, swallowing) [4–7]. Consequently, a decrease in oral function is associated with malnutrition, which is an important outcome factor for recovery and survival rate [8–10]. Semi-regional morbidity can be found in decreased active range of motion (AROM) of shoulders and neck, as well as a decrease in upper body strength [11–13]. The etiology of limitations in neck and shoulder function is multifactorial and lies in a combination of nerve and soft tissue damage and a change in movement patterns due to pain and shoulder disuse mostly related to surgery and radiotherapy [11, 13]. Neck and shoulder problems in sHNC have a high incidence and can pose severe problems during activities in daily life and participation [11].

General morbidity can concern cancer-related fatigue [14], a lower level of physical mobility, and decreased walking ability which limits return to work and daily activities [15]. Treatment related morbidity may be caused by surgery due to resection, reconstruction, neck dissection (ND), by radiotherapy causing fibrosis, skin problems, mucositis, or by systemic responses of chemotherapy [16]. Local, semi-regional and general morbidity lead to a decrease in functional performance in sHNC resulting in limitations in daily activities and difficulty returning to work, which subsequently negatively influences Health-Related Quality of Life (HRQoL) [17, 18]. These findings, together with an increasing number of sHNC, reveal a clear need for rehabilitation interventions focusing on problems in the physical domain. In contrast to this, research shows that sHNC are mostly sedentary (> 50%) and very few participate in moderate or vigorous exercise [15]. However, during treatment 73% of the patients indicated the need for physiotherapy. After 8–11 years, 23% still indicate a need [19].

Several measurements provide insight into the limitations within the physical performance, such as MMO, shoulder and neck mobility, upper and lower body muscle strength, level of mobility, and walking ability. Measurement methods on MMO vary and are performed both intra- and extra-orally [7, 20]. In cancer rehabilitation, a frequently used core set of measurements to objectify physical performance, consists of the measurement of AROM with gonio- or digital inclinometers, grip strength (GS) as proxy for upper body strength, the 30-second chair-to-stand test (30SCTS) for lower body strength, the Timed Up and Go test (TUG) for level of mobility, and the 6-Minute Walk Test (6MWT) for walking ability. This core set of physical performance measurements can be used in addition to Patient Reported Outcome Measurements (PROM’s) on physical status. Insight in test-retest reproducibility of these instruments is important as it illustrates if measurements have the capacity to differentiate between sHNC when measured twice under the same conditions [21]. Insight into agreement parameters is important because it provides information on the Standard Error of Measurement (SEM) and the Smallest Detectable Change (SDC) which are essential for clinical interpretation of the (re)assessment of sHNC. Up to now, this core set of measurements was primarily studied on reliability in other patient populations or included in a case mix of sHNC and HNC patients still undergoing treatment [22–25]. Therefore, this study aims to examine the reliability, by investigating test-retest reproducibility, SEM, and SDC, of a core set of measurements on physical performance in sHNC.

## Methods

### Study setting and participants

Two subgroups participated in this cross-sectional study. Between January and June 2018, the first group of sHNC was recruited by convenience sampling from three regional patient support groups of the Dutch Head and Neck Oncology patient federation (regional support groups: Nijmegen, West-Brabant and Centre of Holland). Between March and June 2019, the second group was recruited from sHNC scheduled for usual care follow-up appointments at the Radboud university medical center. Inclusion criteria were: sHNC, completed medical treatment, 18 years or older, and able to walk unaided.

sHNC that were not able to speak or understand Dutch, patients receiving palliative care, and patients at risk when performing physical measurements were excluded. The safety and possible risk when performing physical measurements was assessed before inclusion, using a modified Physical Activity Readiness Questionnaire (PARQ), leading to the exclusion of willing participants who answered both yes to one or more out of seven questions and were judged on these items by their general practitioner to be unfit or unsafe for exercise [26, 27].

### Sample size calculation

An a-priori sample size calculation was conducted following the recommendations of Donner & Eliasziw [28]. With a more than acceptable intraclass correlation coefficient (ICC) of 0.80, an level of significance of 0.05, and power of 0.8 (β = 0.2) it was established that 45 participants were required in the final analysis. It was anticipated that approximately 10% would drop out for motivational or practical reasons. Thus, the goal became including at least 50 patients in total. This number is sufficient to achieve a score of good on adequate sample size conform the COSMIN checklist [29]. The COSMIN checklist can be used to evaluate the methodological quality of studies on measurement properties of health status measurement instruments.

### Study procedure

Members of the Dutch Head and Neck Oncology patient federation attended a presentation about the research project during a regular federation meeting. If interested, they received the patient information brochure. Before their follow-up appointment, the usual care follow-up group was contacted by telephone to inform about the study and send the patient information brochure. The week following the presentation or phone call, both groups were contacted by telephone to determine if there were any questions and acquire verbal informed consent. Participants then received the PARQ digital questionnaire using Castor (Ciwit BV, Amsterdam, The Netherlands) electronic data capture (EDC) program (http://www.castoredc.com). The measurements took place at the physical therapy department of the Radboud university medical center. Prior to the physical measurements written consent was obtained. The study was conducted according to the principles of the Declaration of Helsinki (64th version, October 19^th^, 2013). The protocol (NL2017-3508) was approved by the Ethics Committee of the Radboud university medical center. This study followed the COSMIN checklist to ensure methodological and statistical quality and reduce bias [29].

### Measurements

The patient's demographic and clinical data including age, sex, body weight, body height, smoking status (yes/ no/ history of smoking, packyears), alcohol usage (yes/no, number of units daily), level of education (lower, middle, higher), social status (living alone, living with partner), years since completion of medical intervention, tumor location (oral cavity, nasopharynx, oropharynx, larynx, other), treatment modality (surgery, radiotherapy, chemotherapy, or combinations of these), and neck dissection status (yes, unilateral/bilateral, no) were obtained using a custom patient reported questionnaire send by the electronic data capture software program Castor (see also Table 1). Measurements were performed in a standardized order and according to a standardized measurement protocol. The MMO was measured using two methods. Method one measured intra-orally with a cardboard ruler (TheraBite^©^ Range of Motion Scale, Atos Medical Inc., New Berlin, Wisconsin, United States). Method two measured MMO extra-orally with a calibrated caliper (Electronic Digital Caliper 150 mm/6”, Somultishop, Echt, Holland) following a previously described protocol [7]. Shoulder abduction and forward flexion were measured with a digital inclinometer (Baseline^©^ Digital Inclinometer, Fabrication Enterprises Inc., White Plains, New York, USA) [30]. External rotation of the shoulder was measured with a goniometer (Universal goniometer, Mathys Synthes, Bettlach, Switzerland). The CROM (Cervical Range of Motion Instrument, Performance Attainment Associates, Lindstrom, Minnesota, USA) was used to measure the lateral flexion and rotation of the neck [31]. Grip strength was measured with a hand-held dynamometer (JAMAR^©^, Sammons Preston Rolyan, Warrenville, Illinois, USA) [32]. The 30SCTS was used to examine lower body strength [33]. The level of mobility was measured with the TUG [24]. Walking ability was evaluated using a self-paced 6MWT on a 20-meter circuit [24].

**Table 1:** Demographic, participant, and treatment characteristics.

Characteristic	Total (n = 50)	SD	PCTL 25^th^ centile; 75^th^ centile	%
Sex				
Male, n	28			56
Female, n	22			44
Age (years), mean	68.6	9.9		
Body Mass index, median	25.0		23.5–26.7	
Smoking				
Yes, n	4			8
Pack-years, median	19		4.0–34.0	
No, but used to, n	39			78
Pack-years in history, median	20		9.0–31.0	
Never, n	7			14
Alcohol usage (>1 daily)				
Yes, n	22			44
Glasses per day, median	2		0.5–3.5	
No, n	28			56
Level of education				
Lower, n (%)	20			40
Middle, n (%)	17			34
Higher, n (%)	13			26
Social status				
Living alone, n (%)	16			32
Living with a partner, n (%)	34			68
Years since cancer treatment, median	3.0		1.0–5.25	
Tumor location				
Oral cavity, n (%)	28			56
Nasopharynx, n (%)	1			2
Oropharynx, n (%)	2			4
Larynx, n (%)	12			24
Other, n (%)	7			14
Oncology treatment				
Surgery, n (%)	19			38
Surgery and radiotherapy, n (%)	18			36
Radiotherapy, n (%)	4			8
Surgery, radiotherapy and chemotherapy, n (%)	7			14
Radiotherapy + chemotherapy, n (%)	2			4
Neck dissection				
Unilateral, n (%)	22			44
Bilateral, n (%)	6			12
No, n (%)	22			44

SD: standard deviation; PCTL: Percentile

Measurements were performed by physical therapy students who received intensive training. Measurements were supervised by an experienced physical therapist. The time interval between the test and retest measurement was at least one hour and maximal two hours. Test and retest were performed by the same physical therapy student. After the first test session, the data collection form was collected by the researcher to limit bias. In accordance with guidelines, during both the test- and retest session the 30SCTS and 6MWT were measured once, MMO and neck and shoulder function were measured twice, and GS and TUG were measured three times. For both test and retest measurement, the best score of each participant was used.

### Statistical analysis

The demographic, personal, and treatment characteristics of the participants were described. Categorical data were presented as exact numbers and percentages were calculated. For the continuous data, means and standard deviations (SD) were calculated. Differences in MMO between the two measurement methods were tested with paired samples t-test in case of normally distributed data or Wilcoxon signed rank test for not normally distributed data. Reliability was divided into test-retest reproducibility and agreement parameters [34]. Test-retest reproducibility was tested using the intraclass correlation coefficient (ICC). ICC’s were calculated using a two-way mixed effect model (ICC3.1_agreement_) with absolute agreement and 95% confidence intervals (CI). Cut-off points for the ICC were chosen as poor (0.01–0.20), slight (0.21–0.51), fair (0.41–0.60), good (0.61–0.80), very good (0.81–0.92), and excellent (0.93–1.00) [35]. Both were expressed in the unit of measurement. SEM was calculated as SEM_agreement_ = √σ2error = √(σ2o+ σ2residual) [36]. The variance due to systematic differences between measurements (σ2o) and the residual variance (σ2residual) was obtained from the varcomp analysis [36]. The SEM_agreement_ was used to calculate the SDC_agreement_ = 1.96 * √n * SEM. In this formula, ‘n’ refers to the number of measurements, which was two in this study. Additionally, the SDC% was calculated as agreement outcome independent of the unit of measurement. The SDC% was calculated by dividing the SDC by the mean of the summed test and retest score, then multiplied by 100. For SDC% a 20% difference was set as cut off value for measurement error being relatively small (<20%) or large (>20%). Bland-Altman plots visualize the relationship between the measurement error and the observed value including the presence of systematic bias and bias related to the magnitude of the test outcome [37]. These plots show the test-retest difference (y-axis) against the mean of the first and second test outcomes (x-axis). Mean differences between the test and retest measurements were calculated with their standard deviations to calculate the 95% limits of agreement (95% LoA). In the plot, 95% LoA are shown (mean difference ± 1.96 * SD of the difference). All analyses were performed using IBM SPSS Statistics v25 (SPSS, Inc., Chicago, Illinois, United States). In all cases, two-sided p-values smaller than 0.05 were considered to be statistically significant.

## Results

**Fig 1:**
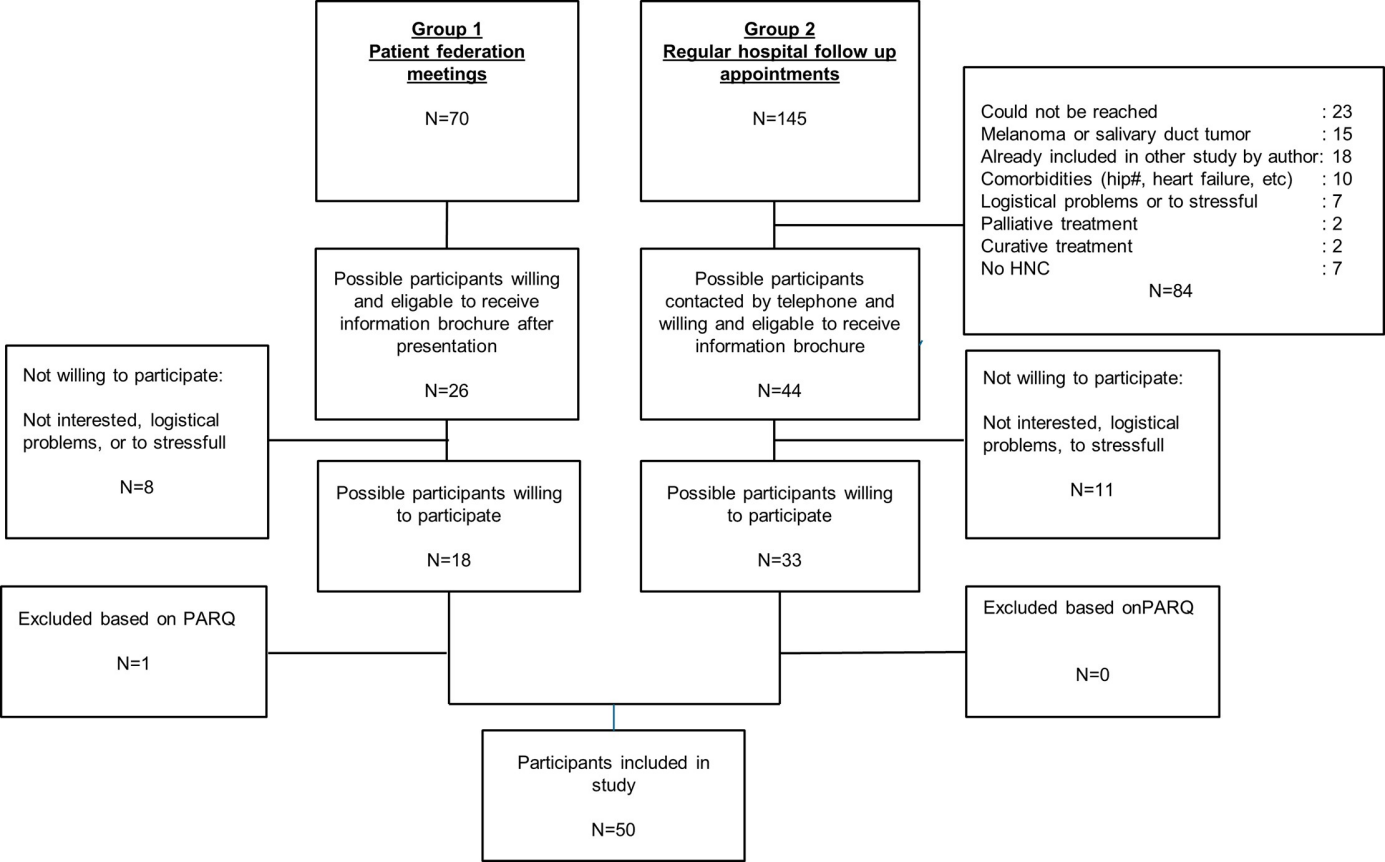
Recruitment and enrollment participants. HNC: Head and Neck Cancer, PARQ: Physical Activity Readiness Questionnaire.

In total 50 sHNC participated in the study, of which 29 were male and 21 were female. Fig 1 shows the flowchart of the recruitment and enrollment of participants. The mean age of participants was 69 years, with a standard deviation of 9.9. The median time of cancer survivorship was 3 years. All demographic, participant, and treatment characteristics are presented in Table 1.

MMO showed no significant difference between the cardboard ruler and the digital caliper at the test measurement (p = 0.08) but MMO measured using the digital caliper was significantly larger (10.1%) at the retest measurement compared to the card ruler (p<0.001).

**Fig 2:**
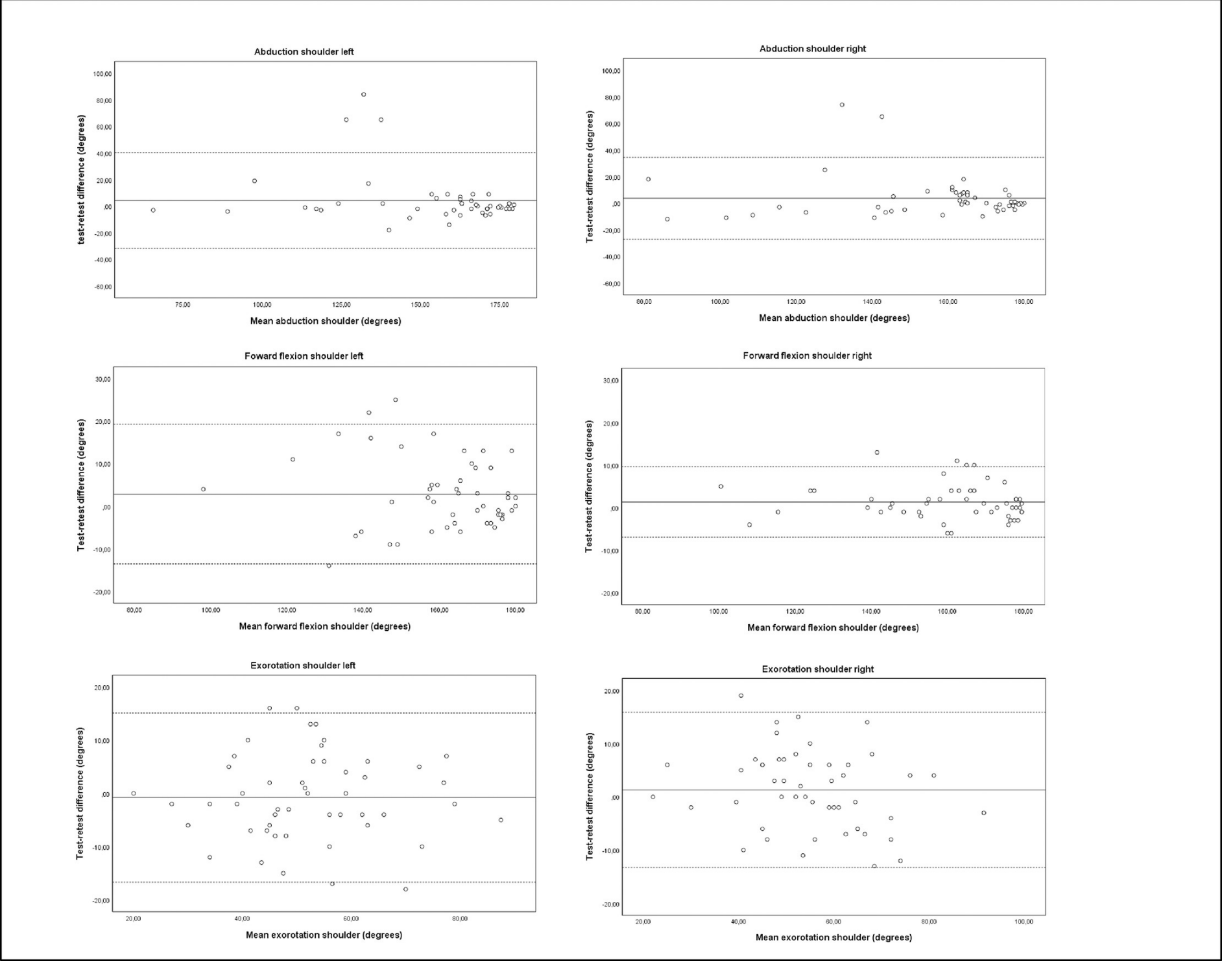
Bland–Altman plots for test-retest reproducibility of maximal mouth opening, shoulder abduction, forward flexion of the shoulder, external rotation of the shoulder. The solid line represents the mean difference (systematic bias) and the dashed lines illustrate the 95% limits of agreement (mean difference ± 1.96 SD of the difference).

**Fig 3:**
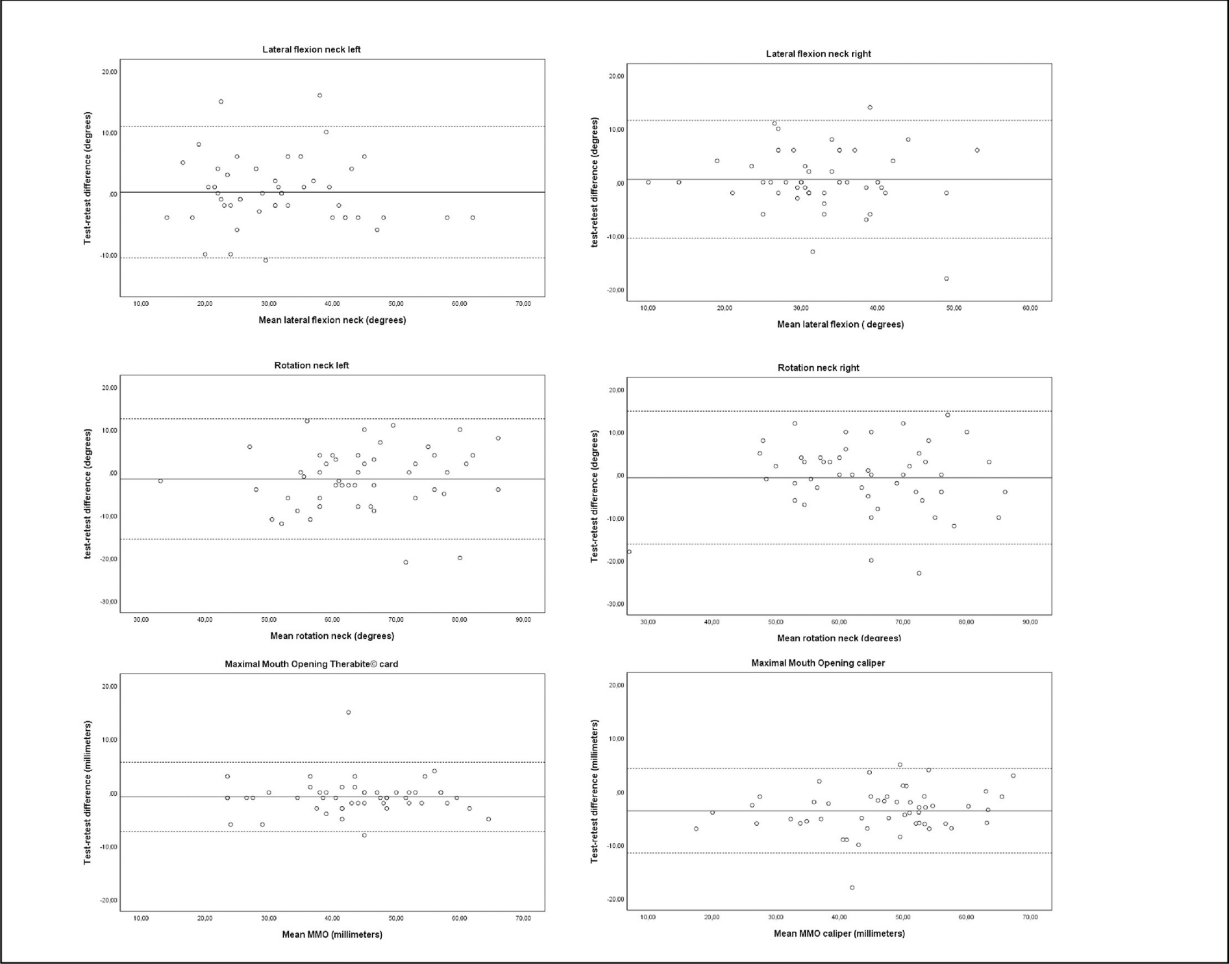
Bland–Altman plots for test-retest reproducibility of lateral flexion of the neck, rotation of the neck, maximal mouth opening. The solid line represents the mean difference (systematic bias) and the dashed lines illustrate the 95% limits of agreement (mean difference ± 1.96 SD of the difference).

**Fig 4:**
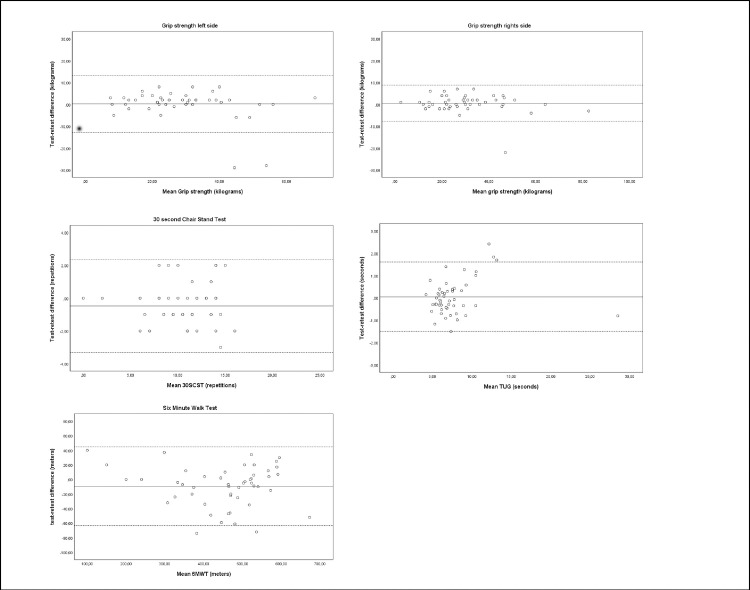
Bland–Altman plots for test-retest reproducibility of grip strength, 30 second chair stand test, Six Minute Walk Test, and Timed Up and Go test. The solid line represents the mean difference (systematic bias) and the dashed lines illustrate the 95% limits of agreement (mean difference ± 1.96 SD of the difference).

The calculated ICC values ranged from 0.77 to 0.98 (see Table 2). These values indicate good to excellent test-retest reproducibility [35]. Agreement expressed in SDC% ranged between 5.4% and 44.7% for the whole core set of physical measurements. MMO measured with cardboard card, forward flexion shoulder and 6MWT had an acceptable measurement error (SDC%: 5.4% - 15.1%) compared to caliper measured MMO, shoulder abduction, shoulder external rotation, later flexion and rotation of the neck, grip strength, 30SCST, and TUG (SDC%: 19.8% - 44.7%). The Limits of Agreement for all measurements are visualized in Figs 2, 3 and 4.

**Table 2:** Reliability of measurements on physical performance in sHNC.

	Test	Retest	Diff test-retest	95% LoA	ICC3.1 (95% CI)	SEM _agreement_**	SDC _agreement_**	SDC %
	Mean (SD)	Mean (SD)	Mean (SD)					
MMO (millimeter)								
Card	43.16 (10.57)	43.98 (10.51)	-0.82 (3.30)	-7.29; 5.65	0.95 (0.91–0.97)*	2.38	6.60	15.1%
Caliper	44.78 (11.90)	48.42 (11.07)	-3.63 (4.03)	-11.53; 4.27	0.90 (0.54–0.96)*	3.81	10.57	22.7%
AROM shoulder forward flexion (degrees)								
Left	162.14 (17.41)	160.16 (17.31)	2.86 (8.38)	-13.56; 19.28	0.95 (0.89–0.97)*	4.08	11.30	7.0%
Right	160.64 (19.48)	159.28 (19.86)	1.36 (4.20)	-6.87; 9.59	0.95 (0.96–0.99)*	3.09	8.57	5.4%
AROM shoulder abduction (degrees)								
Left	156.90 (25.19)	152.62 (29.32)	4.28 (18.40)	-31.79; 40.35	0.77 (0.62–0.86)*	13.23	36.68	23.7%
Right	158.36 (24.97)	154.68 (26.48)	3.68 (15.68)	-27.06; 34.42	0.81 (0.69–0.89)*	11.28	31.27	20.0%
AROM shoulder external rotation (degrees)								
Left	51.88 (14.46)	52.66 (14.48)	-0.78 (8.10)	-16.67; 15.11	0.85 (0.74–0.91)*	5.70	15.80	30.2%
Right	55.82 (13.07)	54.52 (14.83)	1.30 (7.45)	-13.27; 15.87	0.86 (0.76–0.92)*	5.28	14.65	26.6%
AROM neck lateral flexion (degrees)								
Left	31.68 (10.47)	31.50 (10.93)	0.18 (5.48)	-10.56; 10.92	0.87 (0.78–0.93)*	3.84	10.64	33.7%
Right	32.76 (8.41)	32.20 (8.92)	0.56 (5.63)	-10.46; 11.58	0.79 (0.66–0.88)*	3.96	10.97	33.8%
AROM neck rotation (degrees)								
Left	63.86 (11.89)	62.66 (14.48)	-1.52 (7.18)	-15.60; 12.56	0.80 (0.67–0.88)*	5.14	14.25	22.1%
Right	64.26 (12.05)	64.52 (14.83)	-0.66 (7.93)	-16.20; 14.88	0.79 (0.65–0.87)*	5.57	15.44	23.9%
GS (kilogram)								
Left	29.08 (12.73)	28.86 (14.60)	0.22 (6.67)	-12.86; 13.30	0.88 (0.80–0.93)*	4.67	12.96	44.7%
Right	30.20 (14.67)	29.68 (15.42)	0.52 (4.23)	-7.76; 8.80	0.96 (0.93–0.98)*	2.98	8.26	27.6%
30SCST (number of times)	10.56 (3.55)	11.04 (3.90)	-0.48 (1.47)	-3.31; 2.35	0.92 (0.85–0.95)*	1.07	2.96	27.4%
TUG (second)	7.79 (3.69)	7.73 (3.56)	0.05 (0.79)	-1.50; 1.60	0.98 (0.96–0.99)*	0.55	1.54	19.8%
6MWT (meters)	447.18 (117.04)	456.68 (120.12)	-9.50 (27.59)	-63.57; 44.57	0.97 (0.95–0.98)*	20.45	56.67	12.5%

*: p<0.001

**: expressed in unit of measurement; AROM: Active range of motion; CI: confidence interval; ICC: intraclass correlation coefficient; GS: grip strength; LoA: limits of agreement; MMO: maximal mouth opening; SDC: smallest detectable change; SD: standard deviation; SEM: standard error of measurement; TUG: timed up and go; 6MWT: 6-minute walking test; 30SCTS: 30-second chair-to-stand test.

## Discussion

This study establishes good to excellent test-retest reliability of a core set of measurements on physical performance for sHNC in two frequently used measurements on MMO (Therabite^©^ cardboard card (intra orally) and a digital caliper (extra orally)), shoulder and neck AROM, upper body strength (GS), lower body strength (30SCTS), level of mobility (TUG), and walking ability (6MWT). It also provides clinically usable information on measurement error to interpret and evaluate physical performance in sHNC. The measurement error reported in caliper measured MMO, shoulder abduction, shoulder external rotation, lateral flexion of the neck, rotation of the neck, GS, 30SCST and TUG is large in relation to the mean scores of the test and retest measurements. This leads to the question if this variance is related to the testers, the measurement procedure, or the participants. Although measurements were performed by physical therapy students, they received extensive training and supervision during measurements by experienced physical therapists. The measurement protocol was based on guidelines and training sessions were performed to solve possible uncertainties. This advocates that measurement error caused by variance in testers or the measurement procedure should be limited. Possible variance between measurements caused by participants will be discussed per measurement.

MMO measured with the Therabite^©^ cardboard ruler (ICC 0.95) and the digital caliper (ICC 0.90) showed ICC’s that are slightly lower in comparison with measurement of MMO using a normal ruler intra-orally (ICC 0.99) [20]. This however still indicates a good ability to differentiate in MMO between sHNC [20]. Digital caliper scores are systematically higher for the retest measurement compared to the cardboard ruler, indicating more variation in MMO with the digital caliper (Fig 3). One hypothesis for the higher MMO is related to observations made by the students performing the measurements. They observed sHNC experiencing fear of the digital caliper being directly in their field of view during the first test measurement. The participants might have experienced discomfort related to possible contact between the nose or chin and the metal digital caliper. This fear was less present during the retest measurement possibly resulting in a larger MMO. This variation is also illustrated by a higher SEM (3.81 to 2.38), SDC (10.57 to 6.60), and SDC% (22.7% to 15.1%) compared to the Therabite^©^ cardboard ruler. The SEM and the SDC of the Therabite^©^ cardboard are comparable to measurements performed in a population with temporomandibular joint problems (SEM 2.9 and SDC 8.1 mm), providing evaluative values for clinical use. Based on these findings we would favor the use of the Therabite^©^ cardboard ruler in sHNC.

The ICC’s on shoulder abduction, forward flexion and external rotation (ICC 0.77 to 0.95) found in our study are slightly lower than ICC’s measured in healthy subjects (ICC 0.95 to 0.99) With specific problems in shoulder problems to be expected in sHNC, these ICC’s still demonstrate a good ability to differentiate in shoulder function between sHNC [30]. A remarkable finding is the high SDC and SDC% for shoulder abduction and external rotation. Shoulder abduction is an important indicator of accessory nerve damage, associated with a high risk of shoulder pain and limitations in activities in daily life [38, 39]. The high shoulder abduction SDC illustrates a large measurement error between test and retest scores. This measurement error is especially observed in scores on shoulder abduction smaller than 150 degrees (Fig 2). Pain, proprioceptive dysfunction, or decreased upper body strength may have contributed to the use of compensation strategies which could have resulted in confounded measurement results, increasing the measurement error. However, even with extensive training of the testers and the use of a strict measurement protocol, these compensation strategies could not be prevented. This supports clinical examination of the shoulder function by a physical therapist. Future research should take this into account when standardizing measurement protocols.

The ICC’s on neck function measured with CROM device are slightly lower, and the SEM’s are higher compared to literature investigating healthy subjects [31]. This could advert to the CROM device being able to differentiate between sHNC. However, the measurement error is slightly higher compared to healthy subjects when it is used in an evaluative setting. The high SDC% values confirm poor evaluative measurement properties. Variation in measurement outcomes could be related to sHNC undergoing ND surgery and radiotherapy, leading to local alterations in anatomy and physiology causing different compensation strategies [12, 40].

GS ICC scores of 0.96 for the right side and 0.88 for the left side are in line with community-dwelling elderly (right ICC 0.95 and left ICC 0.91), which demonstrates a good ability to differentiate in upper body strength between sHNC [41]. When compared to literature, the SEM for GS was higher (SEM left 4.67, right 2.98) in sHNC compared to healthy individuals (SEM scores for men 2.77, women 1.66). The high SDC% values (45% for the left side, 28% for the right side) illustrate that the measurement error for the GS is too large to be used in a clinical setting which limits evaluative usability [42].

The ICC found for the 30SCST (ICC 0.92) is in line with previous research investigating HNC patients (ICC 0.95) [25] and a study investigating community-dwelling adults (ICC 0.84 men, ICC 0.92 women) [33]. This indicates that the 30SCST can differentiate in functional lower body strength between sHNC. With a mean test-retest score of 11 repetitions and an SDC being nearly 3 repetitions, a sHNC must show an improvement of at least 3 repetitions (SDC% 27%) to be above the measurement error, which limits clinical evaluative usability.

The 6MWT demonstrated an ICC value of 0.97, which is in line with a study that included sHNC and patients with HNC receiving treatment (ICC 0.97). This indicates excellent capability to differentiate in walking ability between sHNC. The SEM of 20.5 meters is lower compared to patients undergoing hemodialysis (SEM 28.4) and comparable to patients with Alzheimer’s, SEM 20.28. The SDC and SDC% indicate that in relation to mean 6MWT test- and retest scores a 13% change is above the measurement error.

Level of mobility was assessed by the TUG which showed a comparable ICC (ICC 0.98) to test-retest studies in people with chronic conditions as Parkinson or stroke [43, 44]. The Bland Altman plot showed homogenous scores for the TUG in our sample (Fig 4). This disputes whether the TUG should be a standard test to differentiate in the level of mobility in sHNC. The SDC score (1.54 sec.) seems relatively small but in percentage (SDC%: 20%) to the average scores (7.73 to 7.79 sec) it is quite large regarding evaluative purposes.

### Strengths and limitations of this study

This study followed the COSMIN checklist to ensure methodological and statistical quality and reduce bias. Similar to other studies, the participants in this study represent a heterogeneous group of sHNC, displaying different characteristics [45–49]. Although specific subgroups in sHNC (for example, patients after laryngectomy) are known to have specific problems in physical performance [50]. The heterogeneity in this sample is likely to provide an adequate representation of the total group of sHNC as found in daily practice. Therefore, this study provides clinically useful information on reliability of a core set of measurements on physical performance.

The selection of participants came from two different groups resulting in a heterogeneous sample of sHNC that improves generalizability. The sHNC contacted through the patient federation had no treatment relationship with the researcher. For this reason, they were asked to report on treatment and tumor characteristics. This allows for mistakes and misinterpretations by the sHNC. The time interval between the test and retest measurement was at least one hour and maximal two hours. Even though intervals of one or two weeks are typically recommended by experts to allow recovery and limit recall bias [51]. The time between the test and retest measurement was chosen because of logistical reasons and was estimated to be long enough to recover from fatigue; the data showed no signs of fatigue. Higher retest measurements were found for both measurements on MMO and 6MWT. This indicates a possible learning effect for these outcomes. This initial learning effect has not been found in previous literature for measurements on MMO and is in line with literature for 6MWT [52]. For all three measurements it does not influence reliability. Another limitation is the absence of measurements on inter-rater reliability. An additional measurement to determine inter-rater reliability was deemed to be too exhausting and time consuming for participants.

### Clinical relevance

More than half of sHNC are sedentary and experience specific problems in physical performance due to treatment of the head and neck area [15]. Insight into reliability of a core set of measurements on physical performance in sHNC is essential to improve supportive care and research on the physical performance of sHNC. To gain full insight into sHNC physical status these measurements can be used in addition to Patient Reported Outcome Measurements (PROMs) that measure patients’ perceptions and views on physical status and performance.

## Conclusion

This study demonstrated good to excellent test- retest reliability of a core set of measurements on physical performance which illustrates that this coreset can be used to differentiate in physical performance between sHNC. The reported measurement errors should be taken into consideration when interpreting the results of repeated measurements.

## Supporting information

S1 File S1 File measurements reproducibility. (SAV)
